# High mortality among patients hospitalized for drug‐resistant tuberculosis with acquired second‐line drug resistance and high HIV prevalence

**DOI:** 10.1111/hiv.13318

**Published:** 2022-05-24

**Authors:** Kim Anderson, Elize Pietersen, Bryan E. Shepherd, Aihua Bian, Keertan Dheda, Robin Warren, Timothy R. Sterling, Yuri F. van der Heijden

**Affiliations:** ^1^ Centre for Lung Infection and Immunity, Division of Pulmonology, Department of Medicine and UCT Lung Institute University of Cape Town Cape Town South Africa; ^2^ Centre for Infectious Disease Epidemiology and Research, School of Public Health and Family Medicine University of Cape Town Cape Town South Africa; ^3^ Department of Biostatistics Vanderbilt University School of Medicine Nashville Tennessee USA; ^4^ South African MRC Centre for the Study of Antimicrobial Resistance University of Cape Town Cape Town South Africa; ^5^ Faculty of Infectious and Tropical Diseases, Department of Infection Biology, London School of Hygiene and Tropical Medicine London UK; ^6^ DST‐NRF Centre of Excellence for Biomedical Tuberculosis Research/SAMRC Centre for TB Research/Division of Molecular Biology and Human Genetics, Faculty of Medicine and Health Sciences Stellenbosch University Tygerberg South Africa; ^7^ Vanderbilt Tuberculosis Center Vanderbilt University School of Medicine Nashville Tennessee USA; ^8^ Division of Infectious Diseases, Department of Medicine Vanderbilt University School of Medicine Nashville Tennessee USA; ^9^ The Aurum Institute Johannesburg South Africa

**Keywords:** acquired resistance, drug‐resistant tuberculosis, HIV, mortality

## Abstract

**Objectives:**

We compared mortality between HIV‐positive and HIV‐negative South African adults with drug‐resistant tuberculosis (DR‐TB) and high incidence of acquired second‐line drug resistance.

**Methods:**

We performed a retrospective review of DR‐TB patients with serial second‐line TB drug susceptibility tests (2008–2015) who were hospitalized at a specialized TB hospital. We used Kaplan–Meier analysis and Cox models to examine associations with mortality.

**Results:**

Of 245 patients, the median age was 33 years, 54% were male and 40% were HIV‐positive, 96% of whom had ever received antiretroviral therapy (ART). At initial drug resistance detection, 99% of patients had resistance to at least rifampicin and isoniazid, and 18% had second‐line drug resistance (fluoroquinolones and/or injectable drugs). At later testing, 88% of patients had acquired additional second‐line drug resistance. Patient‐initiated treatment interruptions (> 2 months) occurred in 47%. Mortality was 79%. Those with HIV had a shorter time to death (*p* = 0.02; log‐rank): median survival time from DR‐TB treatment initiation was 2.44 years [95% confidence interval (CI): 2.09–3.15] versus 3.99 years (95% CI: 3.12–4.75) for HIV‐negative patients. HIV‐positive patients who received ART within 6 months before DR‐TB treatment had a higher mortality hazard than HIV‐negative patients [adjusted hazard ratio (aHR) ratio = 1.82, 95% CI: 1.21–2.74]. By contrast, HIV‐positive patients who did not receive ART within 6 months before DR‐TB treatment did not have a significantly higher mortality hazard than HIV‐negative patients (aHR = 1.09; 95% CI: 0.72–1.65), although those on ART had lower median CD4 counts than those not on ART (157 vs. 281 cells/μL, respectively; *p* = 0.02).

**Conclusions:**

A very high incidence of acquired second‐line drug resistance and high overall mortality were observed, reinforcing the need to reduce the risk of acquired resistance and for more effective treatment.

## INTRODUCTION

Drug‐resistant tuberculosis (DR‐TB) continues to be a public health threat, and treatment success rates for DR‐TB remain unacceptably low.^1^ Although it is expected that wider use of more effective, shorter, all‐oral regimens and the use of patient‐centred models of care will help improve treatment outcomes,^1^ it is important to gain insights from current treatment practices and outcomes to inform successful implementation of these new regimens.

HIV infection appears to increase the risk of DR‐TB, although findings are inconsistent. A meta‐analysis of HIV infection and multidrug‐resistant tuberculosis (MDR‐TB; *Mycobacterium tuberculosis* with resistance to at least isoniazid and rifampicin) found the pooled odds ratio (OR) of MDR‐TB was 1.42‐fold higher in HIV‐positive than in HIV‐negative TB patients [95% confidence interval (CI): 1.17–1.71].^2^ Additionally, HIV infection increases the risk of developing adverse events in DR‐TB patients.^3^ However, several studies have shown similar DR‐TB treatment success rates among patients who are HIV‐negative and HIV‐positive on antiretroviral therapy (ART).^4^, ^5^ A meta‐analysis of mortality in adults with MDR‐TB and HIV found that, compared with HIV‐negative patients, the adjusted OR of death was 2.4 (95% CI: 2.0–2.9) for all patients with HIV‐infection, 1.8 (95% CI: 1.5–2.2) for HIV‐positive patients on ART, and 4.2 (95% CI: 3.0–5.9) for HIV‐positive patients with no or unknown ART.^6^ The impact of HIV is also mixed with regard to worse patterns of anti‐TB drug resistance, including *M. tuberculosis* with resistance to at least isoniazid, rifampicin, any fluoroquinolone and any second‐line injectable drug [previous definition of extensively drug‐resistant TB (XDR‐TB)].^7^, ^8^, ^9^, ^10^, ^11^ Evidence on HIV disease indicators following DR‐TB treatment is scarce, as highlighted in a systematic review of the effects of DR‐TB treatment on HIV disease.^12^

Although transmission of DR‐TB occurs frequently in TB high‐burden settings,^13^ drug resistance can also be acquired spontaneously via *M. tuberculosis* chromosomal mutations followed by anti‐TB drug selection.^14^ HIV was associated with acquisition of rifampicin resistance during treatment for drug‐susceptible TB in a retrospective cohort study of patients in South Africa.^15^ Less is known about the relationship of HIV to acquisition of resistance to fluoroquinolones or second‐line injectable drugs (previously considered second‐line anti‐TB drugs). One multi‐country prospective study of acquired second‐line drug resistance found that HIV was not associated with acquired second‐line drug resistance, although HIV prevalence varied substantially by country.^16^

In this study, we compared DR‐TB treatment outcomes and mortality between HIV‐positive and HIV‐negative adult patients in South Africa who had serial second‐line drug‐susceptibility tests (DSTs) performed.

## METHODS

### Study population

We used National Health Laboratory Service data to identify public‐sector DR‐TB patients in the Western Cape Province who had serial second‐line DSTs performed (primarily ofloxacin and amikacin) between 1 January 2008 and 30 June 2015. Second‐line DSTs were ordered by treating clinicians, suggesting concern for treatment failure and possible drug resistance. Of those identified, we included adults (≥ 18 years at the time of second‐line DST) with known HIV status who were hospitalized at a specialized TB hospital (Brooklyn Chest Hospital) at any time before censor date (30 June 2017). The Brooklyn Chest Hospital provides in‐patient DR‐TB services in Cape Town and surrounding areas. During the study period, recommendations for care of MDR‐TB patients changed from a centralized approach to a decentralized, community‐based care model.^17^ Before decentralization was implemented in 2011, Brooklyn Chest Hospital was the designated DR‐TB treatment hospital that provided in‐patient and outpatient care for most patients with DR‐TB in the Western Cape Province. After decentralization, MDR‐TB patients who did not require hospitalization were treated as outpatients either at Brooklyn Chest Hospital or local clinics, whereas XDR‐TB patients were generally hospitalized. Some study patients were also hospitalized at another specialized TB hospital (DP Marais Hospital). Sources of study data included: (1) detailed medical record reviews of hospital admissions and outpatient visits at Brooklyn Chest and DP Marais Hospitals, (2) laboratory data, (3) mortality data from the Western Cape Provincial Health Data Centre^18^, and (4) mortality data from the National Population Register. We used iterative assessments of approximate matches on patient‐identifying variables to link longitudinal data and data across sources.

For most of the study period, the standardized regimen for MDR‐TB consisted of ≥ 6 months intensive phase treatment with five drugs (kanamycin or amikacin; ofloxacin or moxifloxacin; ethionamide, terizidone and pyrazinamide) followed by continuation‐phase treatment with four drugs (moxifloxacin, ethionamide, terizidone and pyrazinamide).^19^ Patients with resistance to an injectable or a fluoroquinolone required alternative/additional drugs such as capreomycin, para‐amino salicylic acid, moxifloxacin or levofloxacin, high‐dose isoniazid and clofazimine. Bedaquiline was generally unavailable, except for patients enrolled at Brooklyn Chest Hospital in bedaquiline clinical trials^20^, ^21^ or the Bedaquiline Compassionate Access Programme.^22^ TB treatment was directly observed during hospitalization, whereas outpatient treatment was either directly observed or self‐administered.

Patients were considered HIV‐positive if the diagnosis was noted in their medical records. According to South African guidelines, patients with CD4 counts < 200 cells/μL were eligible for ART from 2004; the threshold changed to < 350 cells/μL in 2011 and to < 500 cells/μL in 2015 before universal ART was adopted in 2016.^23^ Guidelines for the management of HIV and TB recommended that those already on ART should continue ART throughout their TB treatment, whereas those not yet on ART should start TB treatment first, followed by ART, usually within 2 months.^24^

### Definitions

Pre‐XDR‐TB was defined as resistance to isoniazid and rifampicin and either a fluoroquinolone or a second‐line injectable drug, but not both. We defined DR‐TB treatment outcomes according to WHO definitions,^25^ with adaptations that specified time ranges around patient sputum collection and allowed outcome determination despite limitations in available data (Table S1). Multiple consecutive outcomes were assigned if there were multiple treatment periods. When assigning treatment outcomes, we categorized patients as MDR, pre‐XDR or XDR, based on DST profile at or nearest (before) the start of treatment. First DR‐TB treatment start date was defined as the first date that a regimen appropriate for MDR‐TB or greater degree of drug resistance was started.^19^ If treatment start date was unknown, we used sputum DST diagnostic date as a proxy. Prior drug‐sensitive and mono‐drug‐resistant TB diagnoses and treatment outcomes were not evaluated. Patients whose most recent treatment outcome at censor date was treatment interruption, lost to follow‐up or not evaluated were re‐evaluated for meeting criteria of clinical failure, which we defined as completing ≥ 8 months of treatment (≥ 243 days) with no overall conversion at the time that treatment ceased.

### Data analysis

We compared characteristics and outcomes of DR‐TB patients living with and without HIV using the χ^2^ test or Fisher's exact test for expected cell frequencies < 5. We described continuous variables using medians and interquartile ranges (IQRs) and used the Wilcoxon rank‐sum test to compare distributions. We used Kaplan–Meier analyses to calculate time to acquisition of resistance and time to mortality from DR‐TB treatment start date and probability of survival by strata (HIV‐positive vs. HIV‐negative at start of DR‐TB treatment). End of follow‐up was date of death or study censor date. Comparisons between strata were made by the log‐rank test. We used multivariable Cox proportional‐hazards models to examine factors associated with mortality. Weibull models were also fitted because of potential violations of the proportional hazards assumptions. Based on the existing literature, we *a priori* selected the following variables for inclusion: HIV status, ART use, sex, age, weight, drug‐resistance profile, alcohol and illicit drug use, calendar year and treatment with bedaquiline. CD4 count was included in analyses limited to HIV‐positive patients. We used covariates and outcome data to multiply impute variables with missing values (25 times) using predictive mean matching. Estimates from the imputation‐specific analyses were then combined using Rubin's rule.^26^ We used restricted cubic splines to model continuous variables. Because missing weight may not have been missing at random, secondary analyses were performed using weight categories including unknown baseline weight. Study data were managed using REDCap hosted at Vanderbilt University.^27^ Analyses were performed using Stata 14 (StataCorp LP; College Station, TX, USA) and R statistical software (v.3.6.3; R Foundation for Statistical Computing, Vienna, Austria). Ethics approvals, including waivers of informed consent, were granted by Vanderbilt University Institutional Review Board (IRB no. 131289) and Human Research Ethics Committees at University of Cape Town (REF 614/2014) and Stellenbosch University (REF N14/08/106). The study was approved by Western Cape Department of Health and City of Cape Town.

## RESULTS

**FIGURE 1:**
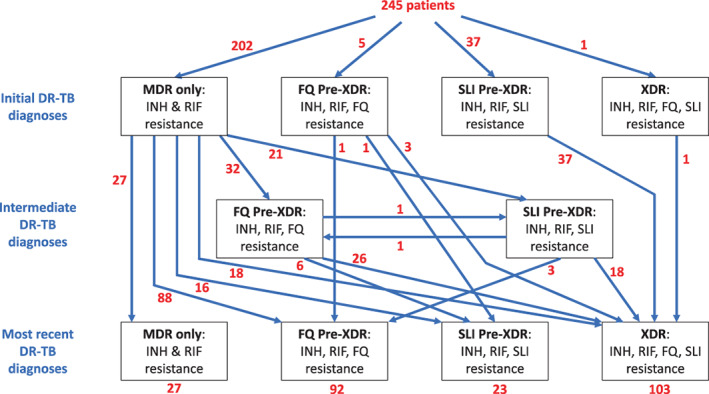
Flow diagram of changing drug‐resistant profiles among the cohort during the study period. Abbreviations: DR‐TB, drug‐resistant tuberculosis; MDR, multidrug resistance (INH isoniazid and RIF rifampicin resistance); FQ, fluoroquinolone; XDR, extensive drug resistance (rifampicin, isoniazid, fluoroquinolone and injectable resistance); SLI, second‐line injectable drugs; FQ Pre‐XDR, isoniazid, rifampicin and fluoroquinolone resistance; SLI Pre‐XDR, isoniazid, rifampicin and injectable resistance. At initial DR‐TB diagnosis, 2 patients with FQ Pre‐XDR did not have rifampicin resistance and 1 patient with XDR did not have isoniazid resistance.

We identified 246 patients (146 HIV‐negative and 99 HIV‐positive; one patient with unknown HIV status was excluded from analysis) who had more than one sputum second‐line DST and were hospitalized at Brooklyn Chest Hospital (Table 1). Initially, 242 (99%) patients had resistance to rifampicin and isoniazid and 43 (18%) had resistance to second‐line drugs (fluoroquinolones and/or injectable drugs) (Figure 1). On subsequent testing, most patients (216/245; 88%) acquired additional second‐line drug resistance to fluoroquinolones and/or injectable drugs; 196 acquired fluoroquinolone resistance and 91 acquired second‐line injectable resistance. Ultimately, 115/245 (47%) patients were diagnosed with pre‐XDR‐TB (47% HIV‐negative vs. 48% HIV‐positive) and 103/245 (42%) with XDR‐TB (43% HIV‐negative vs. 40% HIV‐positive) as their most recent DR‐TB diagnosis during the study period. The median observation time was 3.2 years (3.8 years HIV‐negative vs. 2.7 years HIV‐positive).

**TABLE 1:** Cohort characteristics

	HIV‐negative (*n* = 146)	HIV‐positive (*n* = 99)
Male sex	91 (62.3%)	41 (41.4%)
Age (years) [median (IQR)]^a^	33.0 (24.8–41.9)	33.5 (26.7–38.3)
Weight (kg) [median (IQR)] (*n* = 86; 75)^b^	50.0 (44.7–58.8)	48.3 (42.8–57.5)
Observation time since first DR‐TB treatment start (years) [median (IQR)]^c^	3.8 (2.1–5.5)	2.7 (1.4–4.5)
Total observation time since first DR‐TB treatment start (years)	607.4	330.9
Time from first DR‐TB diagnosis to first DR‐TB treatment start (days) [median (IQR)]	50 (25–93)	56 (30–56)
Total minimum^d^ time on DR‐TB treatment (months) [median (IQR)]^e^	24 (16–34)	21 (12–30)
Greatest degree of drug resistance during study period		
MDR	15 (10.3%)	12 (12.1%)
Pre‐XDR	68 (46.6%)	47 (47.5%)
XDR	63 (43.2%)	40 (40.4%)
Calendar year first DR‐TB treatment initiated		
2003–2006	16 (11.0%)	3 (3.0%)
2007–2010	78 (53.4%)	57 (57.6%)
2011–2014	52 (35.6%)	39 (39.4%)
Total number of TB‐related hospitalizations	329	180
Total number of hospitalizations for TB per patient		
1	49 (33.6%)	48 (48.5%)
2	48 (32.9%)	30 (30.3%)
3	25 (17.1%)	15 (15.2%)
4	13 (8.9%)	4 (4.0%)
≥ 5	11 (7.5%)	2 (2.0%)
Total minimum^d^ time hospitalized per patient (TB‐related) (months) [median (IQR)]	14 (8–19)	12 (8–18)
Ever absconded from hospital	54 (37.0%)	34 (34.3%)
Race		
Mixed ancestry (‘Coloured’)	107 (73.3%)	34 (34.3%)
Black	34 (23.3%)	64 (64.7%)
Other (white and Indian)	5 (3.4%)	1 (1.0%)
Highest level of education attended		
Primary or less	45 (30.8%)	26 (26.3%)
Secondary	88 (60.3%)	61 (61.6%)
Tertiary	6 (4.1%)	3 (3.0%)
Unknown	7 (4.8%)	9 (9.1%)
History of hypertension	8 (5.5%)	1 (1.0%)
History of diabetes	6 (4.1%)	2 (2.0%)
History of chronic kidney disease	4 (2.7%)	3 (3.0%)
History of malignancy	2 (1.4%)	3 (3.0%)
History of epilepsy	4 (2.7%)	0 (0.0%)
History of psychiatric illness	12 (8.2%)	2 (2.0%)
History of imprisonment	30 (20.6%)	11 (11.1%)
History of healthcare work	3 (2.1%)	2 (2.0%)
History of mining work	2 (1.4%)	0 (0.0%)
History of cigarette smoking		
Yes	101 (69.2%)	47 (47.5%)
No	38 (26.0%)	48 (48.5%)
Unknown	7 (4.8%)	4 (4.0%)
History of alcohol use		
Daily	22 (15.1%)	13 (13.1%)
Weekends/‘social’	40 (27.4%)	18 (18.2%)
None	48 (32.9%)	36 (36.4%)
Unknown	36 (24.7%)	32 (32.3%)
History of any illicit substance use	62 (42.5%)	16 (16.2%)
History of marijuana use	48 (32.9%)	11 (11.1%)
History of crystal methamphetamine/methaqualone/cocaine use	41 (28.1%)	14 (14.1%)

Abbreviations: DR‐TB, drug resistant tuberculosis (excluding mono‐drug‐resistant TB); IQR, interquartile range; MDR, multidrug‐resistant tuberculosis; XDR, extensively drug‐resistant tuberculosis.

^a^ At first DR‐TB diagnosis.

^b^ Closest weight to DR‐TB treatment start date within 60 days before or 30 days after.

^c^ First DR‐TB treatment start date was defined as the first date that a regimen appropriate for MDR‐TB or a greater degree of drug resistance was started.

^d^ Excludes periods with unknown start or stop dates.

^e^ Since first DR‐TB diagnosis.

Among 196 patients who acquired ofloxacin resistance, 69 (77%) were HIV‐positive and 127 (82%) were HIV‐negative (*p* = 0.32). Similarly, there was no difference in HIV status among the 91 patients who acquired amikacin resistance or among the 27 patients who had multiple second‐line DST tests but did not acquire ofloxacin or amikacin resistance. Time to acquisition of fluoroquinolone resistance did not differ by HIV status [1.0 year (IQR: 0.6–1.7) HIV‐positive vs. 1.1 years (IQR: 0.5–2.3) HIV‐negative), nor did it differ by lower versus higher CD4 count (< 200 vs. ≥ 200 cells/μL) among patients with HIV.

More than half of patients (60%) had more than one TB‐related hospitalization (52% HIV‐positive vs. 66% HIV‐negative). Median total hospitalization duration per patient was 13 (IQR: 8–19) months. More than a third of patients (36%) terminated a period of hospitalization by absconding from hospital.

There were no significant differences in treatment outcomes stratified by HIV status, including treatment outcomes for the first and most recent DR‐TB treatment periods (Table 2). A substantial proportion of patients experienced at least one patient‐initiated treatment interruption of > 2 months (47%); 18% experienced more than one treatment interruption. Patients whose most recent treatment outcome at censor date was treatment interruption, lost to follow‐up, or not evaluated (36% and 35% of HIV‐negative and HIV‐positive patients, respectively) were re‐evaluated for meeting criteria of clinical failure (completing ≥ 8 months of treatment with no overall culture conversion at the time that treatment ceased). Of these, 48% (25/52 HIV‐negative) and 46% (16/35 HIV‐positive) met the definition of clinical failure. Per treatment outcome definitions, 30% of HIV‐negative versus 38% of HIV‐positive patients died while receiving DR‐TB treatment (*p* = 0.18), whereas at censor date, 77% of HIV‐negative versus 81% of HIV‐positive patients had died overall (*p* = 0.52). Among all study patients, 112 (46%) died during hospitalization. Of the 193 patients who died by censor date, death on treatment was the most recent treatment outcome in 82 (42%) patients. Other treatment outcomes included treatment failure (*n* = 50; 26%), treatment interruption (*n* = 32; 17%), lost to follow‐up (*n* = 4; 2%); cure (*n* = 3; 2%) and not evaluated (*n* = 22; 11%). Among all 23 patients in the cohort whose most recent treatment outcome was cure, four had MDR‐TB, 14 had pre‐XDR‐TB and five had XDR‐TB as the greatest degree of diagnosed drug resistance. Overall, 21 patients (9%) were enrolled in trials/programmes that involved access to bedaquiline (although two patients may have received placebo). Excluding patients who received bedaquiline, mortality was 83% in HIV‐negative (109/13) versus 83% in HIV‐positive (77/93) patients.

**TABLE 2:** Treatment outcomes

	HIV‐negative (*n* = 146)	HIV‐positive (*n* = 99)	*p*‐value
Total number of sequential outcomes^a^ assigned per patient			
1	11 (7.5%)	7 (7.1%)	
2	54 (37.0%)	47 (47.5%)	
3	44 (30.1%)	29 (29.3%)	
4	19 (13.0%)	12 (12.0%)	
≥ 5	18 (12.3%)	4 (4.0%)	
Diagnosis at the start of 1st DR‐TB treatment period			
MDR	122 (83.6%)	74 (74.8%)	
Pre‐XDR	24 (16.4%)	21 (21.2%)	
XDR	0 (0.0%)	4 (4.0%)	
Outcome of first DR‐TB treatment period			0.95
Cure	4 (2.7%)	2 (2.0%)	
Treatment failure	71 (48.6%)	48 (48.5%)	
Death on treatment	4 (2.7%)	3 (3.0%)	
Treatment interruption: early and same treatment resumed	25 (17.1%)	15 (15.2%)	
Treatment interruption: same treatment not resumed	22 (15.1%)	13 (13.1%)	
Not evaluated	20 (13.7%)	18 (18.2%)	
Diagnosis at the start of most recent DR‐TB treatment period			
MDR	32 (21.9%)	23 (23.2%)	
Pre‐XDR	69 (47.3%)	53 (53.5%)	
XDR	45 (30.8%)	23 (23.2%)	
Outcome of most recent DR‐TB treatment period^b^			0.52
Cure	14 (9.6%)	9 (9.1%)	
Treatment failure	36 (24.7%)	17 (17.2%)	
Death on treatment	44 (30.1%)	38 (38.4%)	
Treatment interruption: same treatment not resumed	20 (13.7%)	16 (16.2%)	
Lost to follow‐up	7 (4.8%)	2 (2.0%)	
Not evaluated	25 (17.1%)	17 (17.2%)	
Number of patients with any treatment interruptions^c^	66 (45.2%)	49 (49.5%)	0.51
Number of treatment interruptions^c^ per patient			
0	80 (54.8%)	50 (50.5%)	
1	31 (21.2%)	39 (39.4%)	
2	23 (15.8%)	9 (9.1%)	
≥ 3	12 (8.2%)	1 (1.0%)	
Reason most recent outcome was not evaluated			
Transfer out of province	0 (0.0%)	1 (1.0%)	
Ongoing treatment at study closure	4 (2.7%)	2 (2.0%)	
Insufficient outpatient treatment data	15 (10.3%)	11 (11.1%)	
Nix study – outcome unknown	4 (2.7%)	1 (1.0%)	
Other	2 (1.4%)	2 (2.0%)	
Death by censor date	113 (77.4%)	80 (80.8%)	
Died in hospital^d^ (vs. at home) (*n* = 193)	60 (53.1%)	52 (65.0%)	
Age at death (years) [median (IQR)]	36.0 (27.0–46.2)	35.1 (30.0–40.5)	0.66
Ever had sputum conversion	86 (58.9%)	50 (50.5%)	0.19
If had conversion, ever had sputum reversion (*n* = 86; 50)	71 (82.6%)	41 (82.0%)	0.93
If had conversion, number of reversions per patient (after conversions) (*n* = 86; 50)			
0	15 (17.4%)	9 (18.0%)	
1	50 (58.1%)	25 (50.0%)	
2	16 (18.6%)	16 (32.0%)	
≥ 3	5 (5.8%)	0 (0%)	

Abbreviations: DR‐TB, drug resistant tuberculosis (excluding mono‐drug‐resistant TB); IQR, interquartile range; MDR, multidrug‐resistant tuberculosis; XDR, extensively drug‐resistant tuberculosis.

^a^ Multiple consecutive outcomes were assigned if there were multiple treatment periods.

^b^ For patients who had only one outcome assigned in total, the first outcome is also presented as the most recent outcome.

^c^ In addition to the interruptions tabulated, 10 HIV‐negative and four HIV‐positive patients had treatment interruptions for > 60 days during the continuation phase but the same treatment was resumed for the same diagnosis; such events were not assigned an outcome of treatment interruption, but were regarded as a continuous treatment period.

^d^ Death occurred at any hospital (not restricted to a TB hospital and not restricted to those actively receiving TB treatment or within 7 days of stopping TB treatment).

Most HIV‐positive patients were diagnosed with HIV before DR‐TB treatment initiation (91%) and 70% received ART before or within 8 weeks of DR‐TB treatment initiation (Table 3). The most common ART regimen prescribed during DR‐TB treatment initiation was efavirenz, stavudine and lamivudine. Among patients who ever received ART, 37/95 (39%) had a history of ART non‐adherence for ≥ 30 days. Among patients with available CD4 data prior to DR‐TB treatment start, 36/78 (46%) had CD4 < 200 cells/μL; of those who received any ART within 6 months prior to DR‐TB treatment start, 23/39 of patients (59%) had CD4 < 200 cells/μL compared with 13/39 (33%) not receiving ART (*p* = 0.02). Patients on ART within 6 months prior to DR‐TB treatment versus those not on ART within 6 months had lower pre‐DR‐TB treatment median CD4 counts [157 cells/μL (IQR: 54–303) vs. 281 cells/μL (IQR: 164–400); *p* = 0.02).

**TABLE 3:** Clinical characteristics of HIV‐positive patients (*n* = 99)

Known HIV‐ positive prior to first DR‐TB treatment start date^a^						90/99 (90.9%)	
Ever received ART						95/99 (96.0%)	
On ART within 6 months before DR‐TB treatment start date						45/90 (50.0%)	
If on ART within 6 months before DR‐TB treatment start date, ART regimen used closest to TB treatment initiation				Stavudine + lamivudine + efavirenz		25/45 (55.6%)	
				Zidovudine + lamivudine + efavirenz		7/45 (15.6%)	
				TDF + lamivudine + efavirenz		2/45 (4.4%)	
				TDF + FTC + efavirenz		2/45 (4.4%)	
				Zidovudine + lamivudine + Lopinavir/ritonavir		1/45 (2.2%)	
				Unknown		8/45 (17.8%)	
Known HIV‐positive prior to completing 8 weeks of first DR‐TB treatment						91/99 (91.9%)	
On ART within 8 weeks of first DR‐TB treatment start date						64/91 (70.3%)	
If on ART within 8 weeks of first DR‐TB treatment start date, ART regimen used				Stavudine + lamivudine + efavirenz		46/64 (71.9%)	
				Zidovudine + lamivudine + efavirenz		11/64 (17.2%)	
				Stavudine + lamivudine + nevirapine		2/64 (3.1%)	
				Zidovudine + lamivudine + nevirapine		1/64 (1.6%)	
				Zidovudine + lamivudine + Lopinavir/ritonavir		1/64 (1.6%)	
				Unknown		3/64 (4.7%)	
	**CD4 count (cells/μL) [median (IQR)]**	**CD4 < 200**	**CD4 200–349**	**CD4 ≥ 350**	**VL < 40 copies/mL**	**VL 40–1000 copies/mL**	**VL > 1000 copies/mL**
Pre‐DR‐TB treatment^b^	242 (85–342)	36/78 (46.2%)	23/78 (29.5%)	19/78 (24.4%)	9/17 (52.9%)	3/17 (17.6%)	5/17 (29.4%)
At 1 year post‐DR‐TB treatment start^c^	340 (196–458)	18/70 (25.7%)	18/70 (25.7%)	34/70 (48.6%)	40/66 (60.6%)	11/66 (16.7%)	15/66 (22.7%)
At 2 years post‐DR‐TB treatment start^c^	362 (201–549)	13/54 (24.1%)	13/54 (24.1%)	28/54 (51.9%)	28/48 (58.3%)	6/48 (12.5%)	14/48 (29.2%)
Closest to death date^d^	271 (128–419)	29/78 (37.2%)	18/78 (23.1%)	31/78 (39.7%)	48/73 (65.8%)	10/73 (13.7%)	15/73 (20.6%)

Abbreviations: ART antiretroviral therapy; DR‐TB drug‐resistant tuberculosis (excluding mono‐drug‐resistant TB); FTC, emtricitabine; IQR interquartile range; TDF, tenofovir disoproxil fumarate; VL, viral load (copies/mL).

^a^ First DR‐TB treatment start date was defined as the first date that a regimen appropriate for MDR‐TB or a greater degree of drug resistance was started.

^b^ Closest measurement to treatment start date, within a window of 12 or 6 months before (for CD4 and VL, respectively) and 7 days after.

^c^ Closest measurement to 1 or 2 years after treatment start date, respectively, within a window of 6 months before or after.

^d^ Closest measurement to date of death, within a window of 2 years before.

**FIGURE 2:**
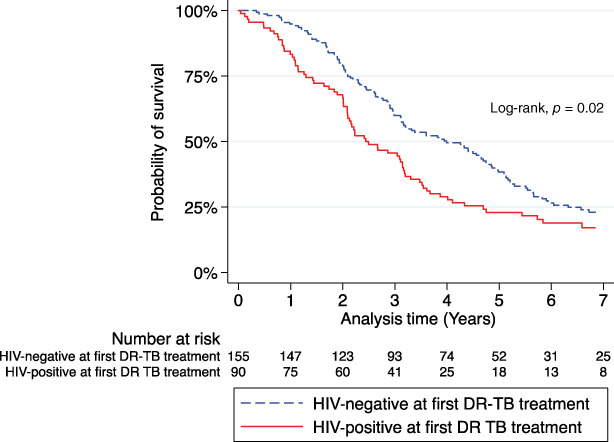
Kaplan–Meier curve showing survival probability since date of first drug‐resistant tuberculosis (DR‐TB) treatment start, stratified by HIV‐negative at DR‐TB treatment start date (or not yet known to be HIV‐positive) versus known HIV‐positive at DR‐TB treatment start date. Figure truncated after 7 years

With Kaplan–Meier analysis (Figure 2), median survival time from first initiation of DR‐TB treatment was 3.99 years (95% CI: 3.12–4.75) for HIV‐negative patients vs. 2.44 years (95% CI: 2.09–3.15) for patients who were HIV‐positive at treatment start. Four‐year survival probability was 0.50 (95% CI: 0.41–0.57) for HIV‐negative patients versus 0.28 (95% CI: 0.19–0.37) for HIV‐positive patients. There was a significant difference in survival between the two groups (*p* = 0.02; log‐rank test).

Cox modelling of time to mortality using multiple imputation of missing baseline weight showed that patients with HIV on ART within 6 months prior to DR‐TB treatment start had a higher mortality hazard compared with HIV‐negative patients [adjusted hazard ratio (aHR) = 1.82, 95% CI: 1.21–2.74] (Table 4). Patients with HIV not on ART within the previous 6 months, however, had no significant difference in time to death (aHR = 1.09, 95% CI: 0.72–1.65). DR‐TB treatment start in earlier calendar years was associated with decreased mortality hazards. Patients age < 30 or > 50 years tended to have higher mortality hazards than those aged 30 years, although the differences for older ages were not significant (Supplementary Figure 1). Receipt of bedaquiline for TB treatment was associated with lower mortality hazards. Weight at DR‐TB treatment start was not associated with mortality in the primary analysis. Weibull model results were similar (Table S2). A secondary analysis using categories of weight (< 50 kg, ≥ 50 kg, unknown) found that baseline weight ≥ 50 kg and unknown weight were both associated with lower mortality hazards than weight < 50 kg (aHR = 0.68, 95% CI: 0.47–0.98 and aHR = 0.64, 95% CI: 0.43–0.94, respectively; Table S3).

**TABLE 4:** Cox model exploring associations between time to mortality and explanatory variables using multiple imputation for missing weight data and restricted cubic splines for continuous variables

Variable	Comparison	Unadjusted analysis			Adjusted analysis		
		HR (95% CI)	*p*‐value	Overall *p*‐value	aHR (95% CI)	*p*‐value	Overall *p*‐value
Age at DR‐TB treatment start (years)	20 vs. 30	1.38 (1.02–1.87)	0.04	0.12	1.46 (1.05–2.03)	0.02	0.06
	40 vs. 30	0.95 (0.83–1.09)	0.47		0.97 (0.84–1.12)	0.67	
	50 vs. 30	1.14 (0.79–1.65)	0.48		1.26 (0.86–1.86)	0.24	
	60 vs. 30	1.40 (0.73–2.71)	0.31		1.71 (0.85–3.41)	0.13	
Weight at DR‐TB treatment start (kg)	40 vs. 50	1.09 (0.76–1.57)	0.63	0.47	1.15 (0.76–1.72)	0.51	0.45
	60 vs. 50	0.89 (0.73–1.10)	0.28		0.88 (0.70–1.11)	0.28	
	70 vs. 50	0.79 (0.47–1.34)	0.39		0.78 (0.44–1.39)	0.40	
Calendar year at DR‐TB treatment start	2005 vs. 2011	0.51 (0.30–0.86)	0.01	0.002	0.49 (0.28–0.85)	0.01	0.001
	2008 vs. 2011	0.70 (0.57–0.86)	0.001		0.59 (0.47–0.74)	<0.001	
	2013 vs. 2011	1.30 (0.95–1.79)	0.10		1.85 (1.31–2.61)	<0.001	
Sex	Female vs. male	1.14 (0.86–1.52)	0.36	0.36	0.90 (0.64–1.26)	0.53	0.53
Bedaquiline part of treatment	Received bedaquiline at any time vs. did not receive bedaquiline	0.33 (0.15–0.69)	0.003	0.004	0.20 (0.09–0.44)	< 0.001	< 0.001
HIV and ART status at DR‐TB treatment start	HIV‐positive on ART vs. HIV‐negative at first DR‐TB treatment	1.67 (1.16–2.40)	0.01	0.02	1.82 (1.21–2.74)	0.004	0.01
	HIV‐positive not on ART vs. HIV‐negative at first DR‐TB treatment	1.23 (0.84–1.80)	0.29		1.09 (0.72–1.65)	0.67	
Prior daily alcohol use (vs. less frequent)	Yes vs. no	0.86 (0.57–1.31)	0.49	0.49	0.82 (0.53–1.26)	0.36	0.36
Prior ‘hard drug’^a^ use (vs. none)	Yes vs. no	1.01 (0.72–1.42)	0.94	0.94	1.11 (0.77–1.59)	0.59	0.59
Drug resistance profile at DR‐TB treatment start	Pre‐XDR and XDR vs. MDR	1.55 (1.09–2.21)	0.01	0.01	1.43 (0.98–2.11)	0.07	0.07

Abbreviations: ART, antiretroviral therapy; aHR, adjusted hazard ratio; CI, confidence interval; DR‐TB, drug‐resistant tuberculosis (excluding mono‐drug‐resistant TB); HR, unadjusted hazard ratio; MDR multidrug‐resistant tuberculosis; XDR extensively drug‐resistant tuberculosis.

^a^ Crystal methamphetamine/methaqualone/cocaine use.

In models restricted to HIV‐positive patients, as CD4 count increased, mortality hazards decreased, although differences were not significant (Table S4). There was no statistically significant difference in mortality hazards between those on ART and those not on ART within 6 months prior to DR‐TB treatment start.

## DISCUSSION

In this group of patients, all of whom were hospitalized at some time during DR‐TB treatment, and most of whom developed worsening TB drug resistance, 79% died by censor date. This supports the notion that ‘the expansion of resistance has ushered in an era of programmatically incurable TB’.^28^ Although mortality during hospitalization (46%) and during treatment (34%) was high, this does not provide a complete picture. With additional follow‐up, many patients who had other unfavourable or unknown treatment outcomes (e.g. treatment failure, treatment interruption, loss to follow‐up, not evaluated) died after the programmatically assigned treatment outcomes. The exceptionally high mortality rate in this highly selected study population suggests the importance of understanding why patients develop additional resistance, particularly so that the same practices are avoided with newer drugs and regimens to treat TB.

We observed a high frequency of patients absconding from hospital (36%) and patient‐initiated treatment interruptions of > 2 months (47%) which are both crude indicators of poor adherence and may have contributed to emerging resistance to second‐line drugs. Most patients (88%) were diagnosed with additional resistance with serial DSTs, suggesting acquisition of additional resistance during treatment, although some patients may have been reinfected with more resistant strains. In a prospective study of MDR‐TB patients without baseline resistance to specific second‐line drugs in 9 countries, 9% of patients acquired XDR‐TB, 11% acquired fluoroquinolone resistance, and 8% acquired resistance to second‐line injectable drugs.^16^ There are several reasons why patients with MDR‐TB may develop additional resistance while receiving treatment: (1) some drugs may not be effective at preventing emergence of resistance; (2) inadequate drug delivery due to poor adherence, inadequate drug dosing, poor or differential absorption can lead to emergence of resistance; and (3) use of standardized regimens without adjustment for baseline drug resistance may result in inadequate regimens.^29^

Overall, HIV‐positive patients had shorter median time to death and a higher probability of death at 4 years. A lower proportion of HIV‐positive patients had recurrent hospitalizations, which is probably due to shorter time to death. After adjusting for potential confounders, patients who were known HIV‐positive and on ART within 6 months before DR‐TB treatment start had a 1.8 times higher mortality hazard compared with patients who were HIV‐negative before DR‐TB treatment start, whereas in patients who were known HIV‐positive but not on ART within the previous 6 months, the association was not significant. The apparent higher mortality risk of ART was probably due to confounding by severity of HIV. Historic CD4 count eligibility criteria for ART access in South Africa meant that those with more advanced HIV disease were more likely to be on ART prior to DR‐TB treatment. This is supported by our finding that median CD4 count of those on ART was significantly lower than those not on ART. Similarly, a study of XDR‐TB patients in two other provinces of South Africa in 2006–2010 found that HIV‐positive patients receiving ART at TB treatment start did not have better outcomes than patients who were not receiving ART.^9^ The authors concluded that ART was given too late in the disease course and that XDR‐TB treatment regimens were insufficient. In our cohort, only 61% of patients with available results were virologically suppressed (HIV viral load < 40 copies/mL) at 1 year post‐DR‐TB treatment start. This is concerning and indicates that these patients are challenging to treat, for TB as well as for HIV.

The strongest associations with mortality, besides HIV and ART status, were calendar period and bedaquiline use. The increased mortality hazard observed in more recent calendar years could be partially explained by the implementation of decentralized care. Only the most ill patients were hospitalized after decentralized care began in 2011 and therefore worse outcomes were observed in hospitalized patients than in earlier calendar periods when all DR‐TB patients were hospitalized. The decreased mortality hazard we observed associated with bedaquiline use is consistent with recent findings that bedaquiline‐based regimens are associated with a large reduction in mortality in DR‐TB patients, compared with standard regimens.^30^ Recent successes achieved with bedaquiline and other new and repurposed drugs require scale‐up if poor global DR‐TB outcomes are to be addressed. However, vigilance for acquisition of resistance to new drugs is necessary.^31^ More extensive drug‐resistance is associated not only with worse outcomes, but also with higher costs of treatment, which in turn limits resource‐limited TB treatment programmes.^32^ The WHO released new guidelines for MDR‐TB treatment in 2019 and 2020 that changed the standard of care and will potentially improve MDR‐TB outcomes through shorter, all‐oral regimens.^33^, ^34^ The high mortality observed in this study reinforces the need for measures to decrease the development of resistance, even with new, more efficacious TB treatment regimens. Evidence of emerging bedaquiline resistance, including among people living with HIV, is concerning and reinforces the need to prioritize the prevention of acquired resistance.^35^

Our study has several limitations. Previous drug‐sensitive and mono‐drug‐resistant TB were not examined, largely due to lack of treatment details available in medical records. Specific DR‐TB regimen details, with the exception of bedaquiline use, and their association with treatment outcomes, including the development of worsening resistance profiles, were not explored but will be studied in future analyses. Detailed ART history was not always well documented in clinical records, therefore associations between poor TB treatment outcomes and ART adherence were not examined. Selection bias is present because we specifically selected patients who had more than one sequential DST and were hospitalized at Brooklyn Chest Hospital, and therefore our cohort is not representative of all DR‐TB cases and these findings cannot be generalized to all DR‐TB patients. Our eligibility criteria excluded patients who remained well enough to be treated as outpatients and those who did not have more than one DST because they had sputum conversion without reversion after starting treatment, and therefore poor outcomes may be overestimated in this cohort compared with all patients with DR‐TB. Other studies of second‐line drug resistance in settings with high HIV prevalence have reported mortality rates ranging from 41% to 98% (XDR‐TB).^7^, ^11^, ^36^, ^37^, ^38^, ^39^ Conversely, patients who did not have more than one DST also include those who died early after diagnosis, before there was time for a second DST (which is usually performed if a patient is still culture‐positive at the end of the intensive phase of treatment) and possibly even before referral for treatment and/or hospitalization. This survival bias could cause an underestimation of poor outcomes. A previous South African study of patients with HIV demonstrated that mortality was highest in the first 30 days after sputum collection, with 40% of patients with MDR‐TB and 51% of patients with XDR‐TB dying during this time.^40^ Similarly, another study showed that 24% of patients with XDR‐TB died before diagnostic results were available and appropriate treatment started.^7^ Study strengths include: (1) patients were not selected by HIV status and therefore reflect real‐world patients in a high‐HIV‐prevalence setting who had evolving DR‐TB disease, changing DST profiles, complicated treatment histories and required hospitalization; (2) we observed a long duration of follow‐up, reflecting more real‐world outcomes than in clinical trials; and (3) multiple sources of mortality data greatly improved our ability to report death.

## CONCLUSIONS

Very high incidence of acquired second‐line drug resistance and high overall mortality were observed during and after hospitalization in adult DR‐TB patients who had multiple second‐line DSTs performed. These findings reinforce the need to reduce treatment interruptions and resistance acquisition during treatment, both in widely used anti‐TB drugs and regimens reflected in this study and in anticipation of newer, more efficacious drugs and regimens.

## AUTHOR CONTRIBUTIONS

Study conception: YFvdH; study design: YFvdH, EP and KA; data collection: EP and KA; data analysis: KA and AB; writing of manuscript draft: KA. All authors reviewed and approved the manuscript before submission.

## CONFLICT OF INTEREST

KA received funding from Viiv Healthcare which is not related to this project. All other authors declare there are no conflicts of interest.

## Supporting information


**Appendix S1** Supporting Information.
