# Supplementary material for: High mortality among patients hospitalized for drug‐resistant tuberculosis with acquired second‐line drug resistance and high HIV prevalence
Source: HIV Med. 2022 May 24;23(10):1085–97. doi: 10.1111/hiv.13318 (PMC9588462; doi:10.1111/hiv.13318)
Supplement: Supplementary file 1 — Appendix S1 Supporting Information. [file HIV-23-1085-s001.docx]

**Supplementary material**

**Table S1: Drug-resistant tuberculosis treatment outcome definitions,** **adapted from World Health Organization 2013 definitions**

| Cure | Treatment completion, without evidence of treatment failure, and with at least 3 consecutive negative sputum cultures (taken ≥23 days apart) after the intensive† phase and without subsequent positive sputum cultures in the treatment period. |
| --- | --- |
| Treatment completion | Completion of programmatic treatment, as recommended by the national TB programme, without evidence of treatment failure, but with no record of at least 3 consecutive negative sputum cultures (taken ≥23 days apart) after the intensive phase. If no specific documentation was available that the intended treatment course was completed, we regarded completion as a minimum treatment duration of 18 months. (This applied to bedaquiline-containing regimens too, with the exception of Nix-TB trial participants; minimum treatment duration of 6 months applied.) |
| Treatment failure | Treatment termination or the need for permanent regimen change of at least 2 anti-TB drugs because of: (a) lack of conversion‡ by the end of the intensive phase, or (b) bacterial reversion§ in the continuation phase after conversion to negative, or (c) evidence of additional acquired resistance to fluoroquinolones or second-line injectable drugs, or (d) adverse drug reactions. (Treatment termination is provider-initiated and was defined as no new regimen starting within ≤7 days of drugs being stopped, whereas regimen change was defined as a new regimen starting within ≤7 days of drugs being changed/stopped.) |
| Treatment interruption | Patient-initiated cessation of treatment which was either: (a) treatment interruption for >2 consecutive months (>60 days) during the intensive or continuation phase; and the same treatment for the same diagnosis was not restarted, but the patient remained traceable by hospital records or laboratory records (same treatment was defined as all drugs in the regimen remained the same plus or minus one drug added or switched; a change from one fluoroquinolone to another was not counted towards the number of drugs changed/switched, nor was a change from kanamycin to amikacin or vice versa) OR (b) treatment interruption for >2 consecutive months (>60 days) during the intensive phase, but the same treatment was resumed for the same diagnosis (i.e. early treatment stopped but resumed). (When such patients restarted treatment, it constituted a new treatment period. However, if treatment was interrupted for >60 days during the continuation phase but the same treatment was resumed for the same diagnosis, this was not assigned an outcome of treatment interruption, but was regarded as a continuous treatment period.) |
| Loss to follow-up | Treatment interruption for >2 consecutive months (>60 days), treatment not restarted and patient untraceable (by hospital records or laboratory records). |
| Death | Death, for any reason, while on TB treatment, within ≤7 days of a known treatment stop date, or within ≤37 days of a presumed¶ treatment stop date. Death supersedes any other treatment outcome at that time point. |
| Not evaluated | This outcome was assigned if unable to assign any of the options above. This included, but was not limited to, patients with missing clinical data, patients with ongoing treatment (receiving treatment for TB at the study censor date) and patients who transferred out to another province for TB care. |

† Intensive phase of treatment was regarded as the first 8 months from treatment initiation; continuation phase as the period thereafter.

‡ We defined conversion as 2 consecutive negative sputum cultures, taken ≥23 days and ≤120 days apart. The first negative culture date was used as the date of conversion. ‘Lack of conversion’ required ≥60 days of treatment and at least two positive cultures since the start of treatment.

§ We defined reversion as 2 consecutive positive sputum cultures, after conversion, taken ≥23 days and ≤120 days apart. The first positive culture date following conversion was used as the date of reversion.

¶ For periods when TB treatment history was incomplete, treatment was ‘presumed’ to have continued if there were at least 2 indicators of care during the period: (1) regular sputum tests (we regarded sputum results ≥75 days apart as an indication of treatment interruption in the absence of detailed treatment history); (2) evidence of hospitalisation; (3) evidence of outpatient treatment; or (4) indications that treatment occurred in transitions between outpatient and inpatient periods.

**Table S2:** **Weibull model exploring associations between survival time and explanatory variables using multiple imputation for missing weight data and restricted cubic splines for continuous variables. Weibull model picked due to concerns about violation of the proportionality assumption in Cox models.**

| **Variable** | **Comparison** | **aETR (95% CI)** | **p-value** | **Overall p-value** |
| --- | --- | --- | --- | --- |
| Age at DR-TB treatment start (years) | 20 vs. 30 | 0.75 (0.60-0.94) | 0.01 | 0.04 |
|  | 40 vs. 30 | 1.04 (0.94-1.15) | 0.46 |  |
|  | 50 vs. 30 | 0.87 (0.67-1.14) | 0.30 |  |
|  | 60 vs. 30 | 0.71 (0.44-1.14) | 0.16 |  |
| Weight at DR-TB treatment start (kg) | 40 vs. 50 | 0.92 (0.69-1.22) | 0.56 | 0.57 |
|  | 60 vs. 50 | 1.08 (0.92-1.26) | 0.38 |  |
|  | 70 vs. 50 | 1.15 (0.77-1.72) | 0.49 |  |
| Calendar year at DR-TB treatment start | 2005 vs. 2011 | 1.75 (1.20-2.54) | 0.004 | 0.001 |
|  | 2008 vs. 2011 | 1.48 (1.28-1.72) | <0.001 |  |
|  | 2013 vs. 2011 | 0.64 (0.51-0.80) | <0.001 |  |
| Sex | Female vs. Male | 1.09 (0.86-1.38) | 0.48 | 0.48 |
| Bedaquiline part of treatment | Received bedaquiline at any time vs. Did not receive bedaquiline | 3.09 (1.78-5.37) | <0.001 | <0.001 |
| HIV and ART status at DR-TB treatment start | HIV-positive on ART vs. HIV-negative at first DR-TB treatment | 0.66 (0.50-0.88) | 0.004 | 0.02 |
|  | HIV-positive not on ART vs. HIV-negative at first DR-TB treatment | 0.93 (0.70-1.24) | 0.62 |  |
| Prior daily alcohol use (vs. less frequent) | Yes vs. No | 1.15 (0.86-1.55) | 0.35 | 0.35 |
| Prior ‘hard drug’† use (vs. none) | Yes vs. No | 0.93 (0.73-1.20) | 0.59 | 0.60 |
| Drug Resistance Profile at DR-TB treatment start | Pre-XDR and XDR vs. MDR | 0.80 (0.61-1.04) | 0.10 | 0.10 |

Abbreviations: aETR adjusted event time ratio; DR-TB drug-resistant tuberculosis (excluding mono-drug-resistant TB); MDR multidrug-resistant tuberculosis; XDR extensively-drug-resistant tuberculosis; ART antiretroviral therapy

† crystal methamphetamine/methaqualone/cocaine use

**Table S3: Cox model exploring associations between time-to-mortality and explanatory variables using restricted cubic splines for continuous variables (secondary analysis without multiple imputation for missing weight data)**

| **Variable** | **Comparison** | **aHR (95% CI)** | **p-value** | **Overall p-value** |
| --- | --- | --- | --- | --- |
| Age at DR-TB treatment start (years) | 20 vs. 30 | 1.42 (1.03-1.96) | 0.03 | 0.06 |
|  | 40 vs. 30 | 0.99 (0.86-1.15) | 0.93 |  |
|  | 50 vs. 30 | 1.32 (0.90-1.94) | 0.15 |  |
|  | 60 vs. 30 | 1.83 (0.93-3.60) | 0.08 |  |
| Weight at DR-TB treatment start (kg) | ≥50 vs. <50 | 0.68 (0.47-0.98) | 0.04 | 0.04 |
|  | Unknown† vs. <50 | 0.64 (0.43-0.94) | 0.02 |  |
| Calendar year at DR-TB treatment start | 2005 vs. 2011 | 0.54 (0.31-0.93) | 0.03 | 0.001 |
|  | 2008 vs. 2011 | 0.59 (0.48-0.74) | <0.001 |  |
|  | 2013 vs. 2011 | 2.00 (1.40-2.86) | <0.001 |  |
| Sex | Female vs. Male | 0.91 (0.66-1.26) | 0.57 | 0.57 |
| Bedaquiline part of treatment | Received bedaquiline at any time vs. Did not receive bedaquiline | 0.19 (0.09-0.43) | <0.001 | <0.001 |
| HIV and ART status at DR-TB treatment start | HIV-positive on ART vs. HIV-negative at first DR-TB treatment | 1.71 (1.14-2.58) | 0.01 | 0.04 |
|  | HIV-positive not on ART vs. HIV-negative at first DR-TB treatment | 1.10 (0.73-1.66) | 0.64 |  |
| Prior daily alcohol use (vs. less frequent) | Yes vs. No | 0.82 (0.54-1.26) | 0.37 | 0.37 |
| Prior ‘hard drug’‡ use (vs. none) | Yes vs. No | 1.15 (0.80-1.65) | 0.44 | 0.44 |
| Drug Resistance Profile at DR-TB treatment start | Pre-XDR and XDR vs. MDR | 1.42 (0.96-2.09) | 0.08 | 0.08 |

Abbreviations: aHR adjusted hazard ratio; DR-TB drug-resistant tuberculosis (excluding mono-drug-resistant TB); MDR multidrug-resistant tuberculosis; XDR extensively-drug-resistant tuberculosis; ART antiretroviral therapy

† Weights were generally available in the medical records of patients who started treatment in hospital, whereas they were often not available for those who started treatment as outpatients at clinics.

‡ crystal methamphetamine/methaqualone/cocaine use

**Table S4: Cox proportional hazard model of time-to-mortality and associations with explanatory variables among HIV-positive patients**

| **Variable** | **Comparison** | **aHR (95% CI)** | **p-value** | **Overall p-value** |
| --- | --- | --- | --- | --- |
| Age at DR-TB treatment start (years) | 20 vs. 30 | 1.60 (0.81- 3.19) | 0.18 | 0.32 |
|  | 40 vs. 30 | 1.01 (0.71- 1.43) | 0.97 |  |
|  | 50 vs. 30 | 1.58 (0.61- 4.10) | 0.35 |  |
|  | 60 vs. 30 | 2.62 (0.48-14.41) | 0.27 |  |
| Weight at DR-TB treatment start (kg) | 40 vs. 50 | 0.90 (0.48-1.72) | 0.76 | 0.76 |
|  | 60 vs. 50 | 0.91 (0.63-1.32) | 0.62 |  |
|  | 70 vs. 50 | 0.72 (0.28-1.83) | 0.49 |  |
| Calendar year at DR-TB treatment start | 2005 vs. 2011 | 0.58 (0.12-2.90) | 0.51 | 0.01 |
|  | 2008 vs. 2011 | 0.62 (0.36-1.06) | 0.08 |  |
|  | 2013 vs. 2011 | 2.12 (1.17-3.85) | 0.01 |  |
| CD4 count **(**cells/µl) | 200 vs. 100 | 1.04 (0.73-1.48) | 0.82 | 0.08 |
|  | 250 vs. 100 | 1.01 (0.63-1.64) | 0.96 |  |
|  | 350 vs. 100 | 0.85 (0.44-1.61) | 0.61 |  |
|  | 500 vs. 100 | 0.50 (0.21-1.20) | 0.12 |  |
| Sex | Female vs. Male | 1.21 (0.66-2.22) | 0.53 | 0.53 |
| ART status at DR-TB treatment start | HIV-positive not on ART vs. HIV-positive on ART | 0.80 (0.46-1.40) | 0.44 | 0.44 |
| Drug Resistance Profile at DR-TB treatment start | Pre-XDR and XDR vs. MDR | 1.68 (0.96-2.92) | 0.07 | 0.07 |

Abbreviations: aHR adjusted hazard ratio; DR-TB drug-resistant tuberculosis (excluding mono-drug-resistant TB); MDR multidrug-resistant tuberculosis; XDR extensively-drug-resistant tuberculosis; ART antiretroviral therapy

**Figure S1: Association between baseline (at drug-resistant tuberculosis treatment start) continuous covariates (A: calendar year; B: age; C: weight; D: CD4 count) and time to death using multiple imputation for missing weight values. A, B, C from models with all patients; D from model restricted to HIV-positive patients only.**

**A) Calendar year**

**
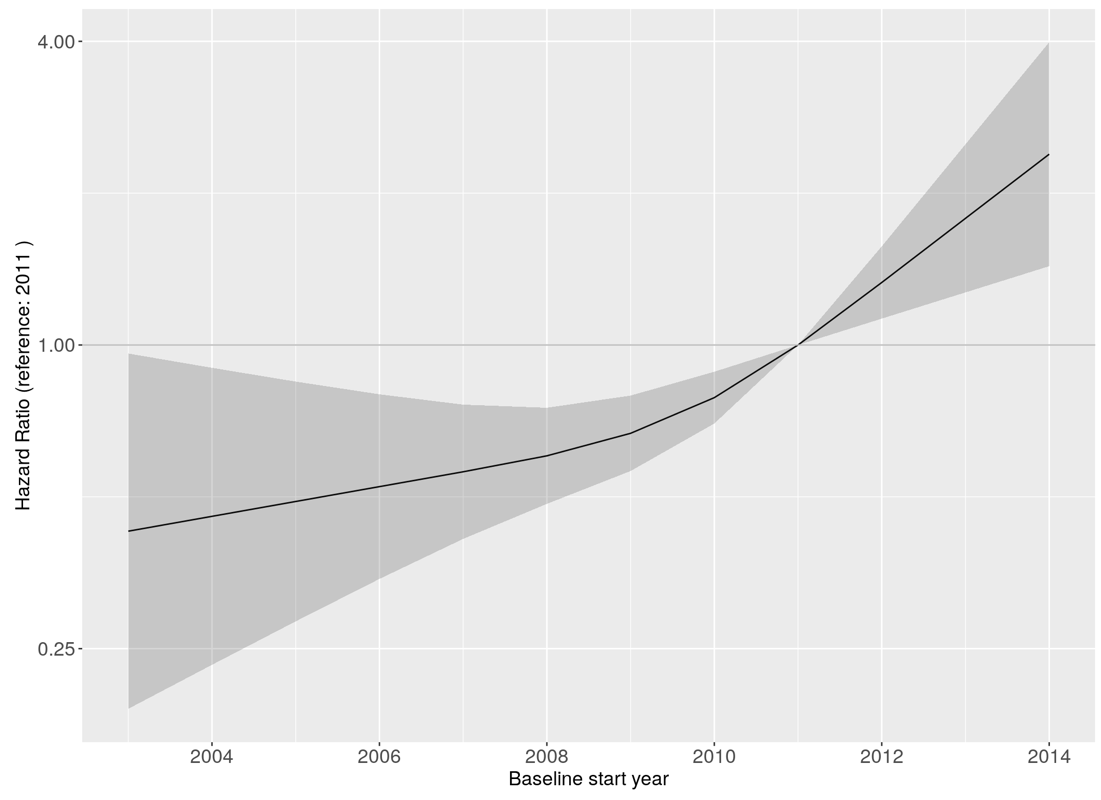
**

**B) Age (years)**

**
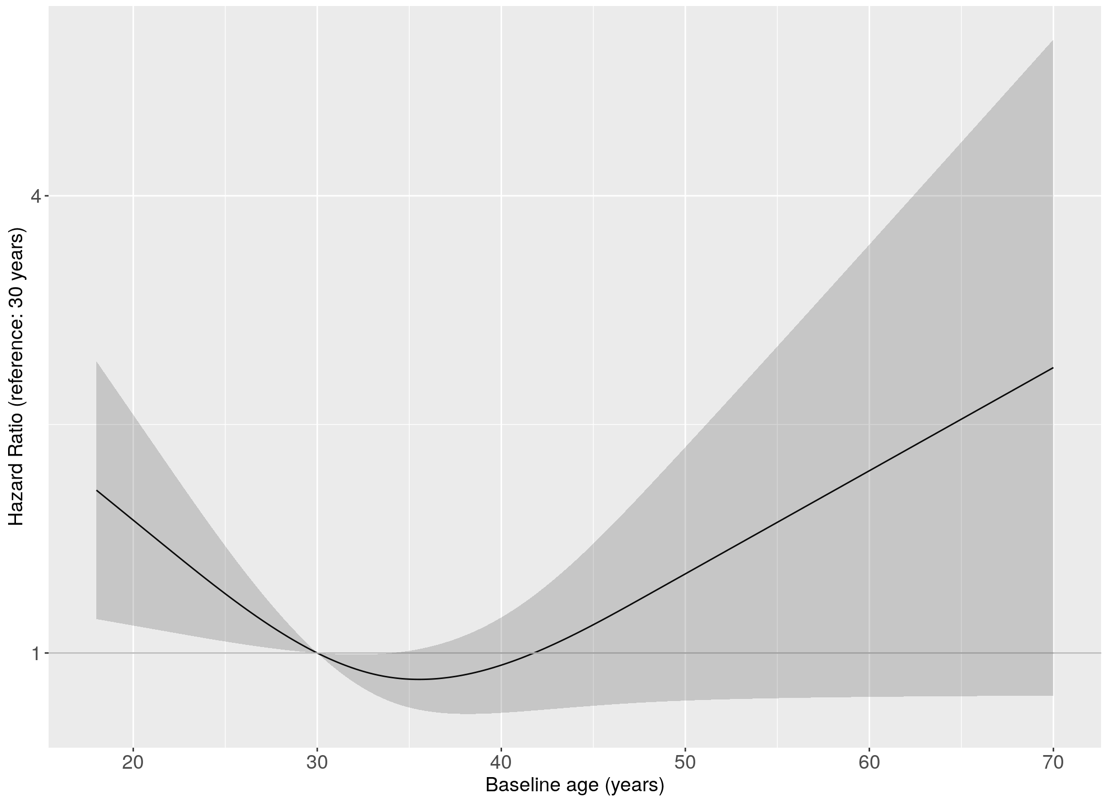
**

**C) Weight (kilograms)**

**
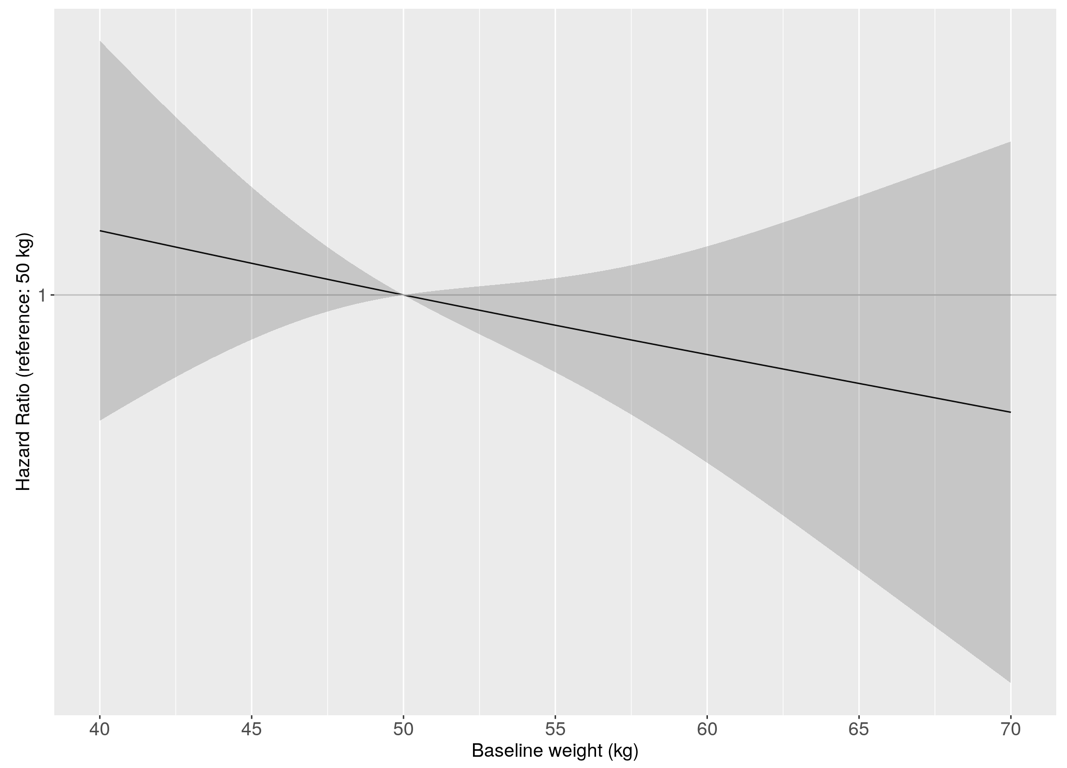
**

**D) CD4 counts (cells/µl)**

**
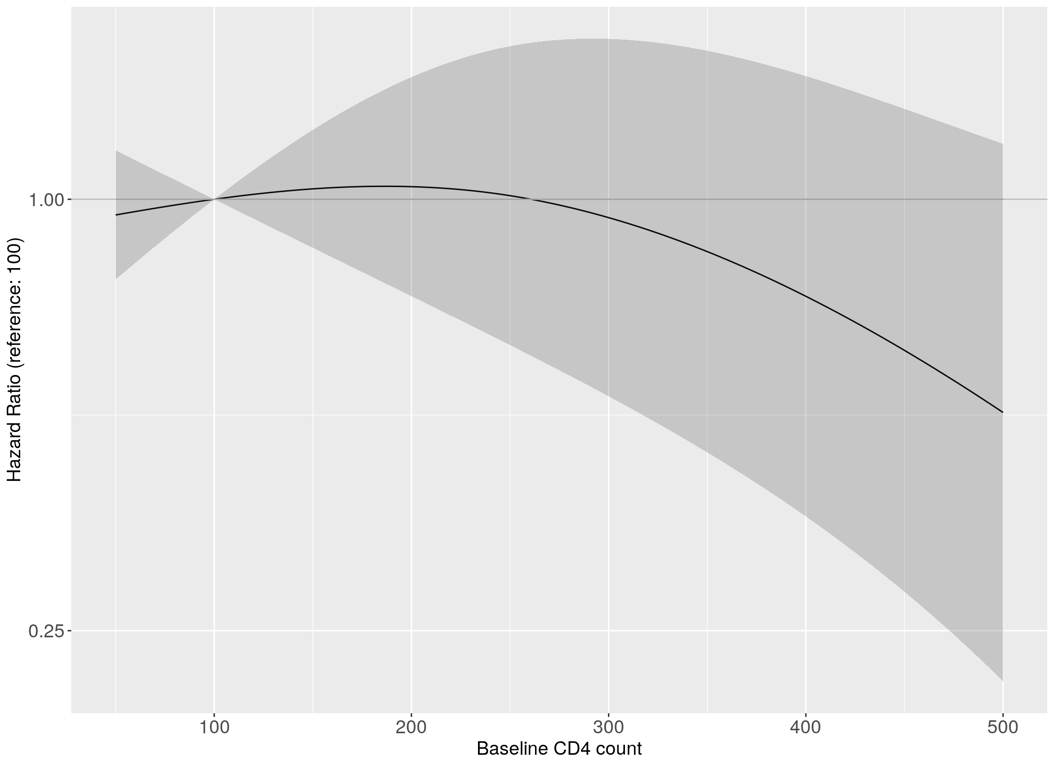
**
